# A Promising Therapeutic Strategy of Combining Acoustically Stimulated Nanobubbles and Existing Cancer Treatments

**DOI:** 10.3390/cancers16183181

**Published:** 2024-09-17

**Authors:** Deepa Sharma, Tera N. Petchiny, Gregory J. Czarnota

**Affiliations:** 1 Physical Sciences, Sunnybrook Research Institute, Toronto, ON M4N 3M5, Canada; tera.petchiny@sri.utoronto.ca; 2 Department of Radiation Oncology, Sunnybrook Health Sciences Centre, Toronto, ON M4N 3M5, Canada; 3 Departments of Medical Biophysics, and Radiation Oncology, University of Toronto, Toronto, ON M4N 3M5, Canada

**Keywords:** cancer treatment, endothelial cell death, microbubbles, nanobubbles, vasculature damage, ultrasound

## Abstract

**Simple Summary:**

Submicron bubbles, known as nanobubbles, are demonstrated to overcome the limitations of microbubbles. The small size of the bubbles allows it to penetrate the vascular endothelial wall, enabling it to move freely through tumor tissues. Studies suggest that ultrasound-stimulated nanobubbles (USNBs) enhance the effect of existing cancer therapies (chemotherapy, immunotherapy, and/ or radiation therapy). This review overviews recent research on nanobubbles and their combinatory biological effects with existing cancer therapies.

**Abstract:**

In recent years, ultrasound-stimulated microbubbles (USMBs) have gained great attention because of their wide theranostic applications. However, due to their micro-size, reaching the targeted site remains a challenge. At present, ultrasound-stimulated nanobubbles (USNBs) have attracted particular interest, and their small size allows them to extravasate easily in the blood vessels penetrating deeper into the tumor vasculature. Incorporating USNBs with existing cancer therapies such as chemotherapy, immunotherapy, and/or radiation therapy in several preclinical models has been demonstrated to have a profound effect on solid tumors. In this review, we provide an understanding of the composition and formation of nanobubbles (NBs), followed by the recent progress of the therapeutic combinatory effect of USNBs and other cancer therapies in cancer treatment.

## 1. Introduction and Backgrounds

**Figure 1:**
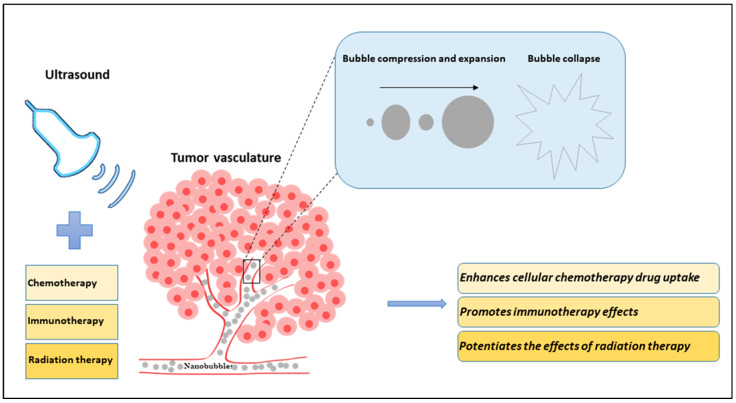
Diagram illustrating the effects of nanobubbles (NBs) and ultrasound in combination with existing cancer therapies (chemotherapy, immunotherapy, and radiation therapy) on tumor vasculature. In an in vivo model, NBs are systemically injected into the blood vessels using a tail vein catheter. Ultrasound is applied to stimulate the NBs, causing them to compress and expand in a violent manner and ultimately causing them to collapse. The tiny bubbles can enhance the vessel permeability and can extravasate the blood vessel inducing mechanical tumor vessel disruption. The sensitized or damaged vessels increase the effectiveness of cancer treatment. The combined treatment of ultrasound-stimulated nanobubbles (USNBs) and cancer therapies is known to improve tumor response.

Ultrasound-targeted microbubble destruction has proven to be an innovative method for enhancing cancer treatment response and non-invasive delivery of drugs/genes [1,2,3,4]. When bubbles come in contact with ultrasound, they start vibrating and oscillating, ultimately causing the bubbles to collapse [5]. Depending on the pressure applied, bubbles undergo different phenomena. Low ultrasound pressure causes bubbles to oscillate, resulting in a phenomenon known as stable cavitation [6,7]. Higher energy deposition causes bubbles to undergo inertial cavitation causing bubbles to rupture, resulting in tiny bubble fragments [8,9,10]. The combination of ultrasound-stimulated microbubbles (USMBs) induces perforation of the endothelial lining of tumor blood vessels. This leads to disruption of tumor vasculature, ultimately causing secondary tumor cell death. The process of vascular endothelial damage is known to be dependent on the activation of the acid sphingomyelinase (ASMase)/ceramide pathway [11,12]. Recently, a new class of submicron bubbles “nanobubbles (NBs)” has been shown to further maximize the therapeutic response upon cancer treatment [13]. NBs are tiny bubbles ranging from less than 500 nm in diameter. They are composed of either protein, polymer, or lipid and stabilized within a biocompatible material [14,15,16] (details provided in next section). NBs can vary in size and material composition depending on the intended function [14,17,18,19]. They are stable, non-buoyant nanoparticles that are non-hemolytic and non-toxic in vivo [14,17,19,20,21,22,23,24,25]. NBs can be used as contrast agents or adapted to transport various forms of protein, DNA, RNA, gas, and therapeutic compounds for targeted delivery [14,18,19,20,22,23,24,26,27,28,29,30,31,32]. NBs have diverse applications in both therapeutic and diagnostic paradigms [14,18,19,20,21,23,24,29]. NBs possess certain advantages over microbubbles. Due to the small size of NBs, they can easily extravasate the perivascular space and penetrate deeper into tissue regions/tumor vasculature as compared to microbubbles that have difficulty reaching the treatment target site [31,33]. Additionally, they can remain in circulation for a longer time with greater stability as compared to microbubbles [31,34]. NBs have proven to be safe and noninvasive [35]. These bubbles are multifunctional as they can be used for both diagnostic as well as theranostic purposes [14]. In recent years, several preclinical studies have reported the successful use of USNBs as an adjuvant therapy alongside other cancer therapies for enhancing the therapeutic efficacy of cancer treatment [13,36,37] (Figure 1). To date, there are a myriad of preclinical studies combining USNBs with chemotherapy; however, there are limited studies of USNBs combined with radiation therapy and/or immunotherapy.

## 2. Structure and Composition of Nanobubbles

**Figure 2:**
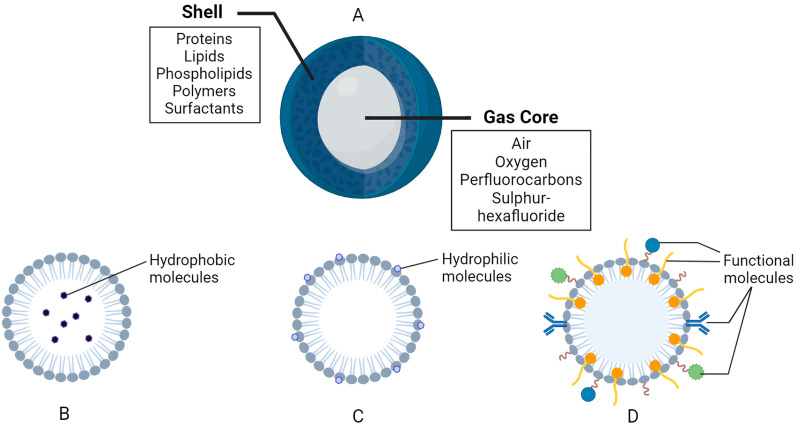
General composition of nanobubbles (NBs). (**A**) General structure and composition. (**B**) Example of a hydrophobic region in a lipid-based shell. (**C**) Example of a hydrophilic region in a lipid-based shell. (**D**) Functional molecules, such as polymers, surfactants, antibodies, binding ligands, etc., attached and/or embedded in the shell membrane. The figure was created with BioRender.com.

NBs are ultrafine spheres that range from 10 to 500 nm in diameter. They comprise two primary components, the inner layer, known as the gas core, and the outer layer or shell [14,17,18,19,20,21,28] (Figure 2). The gas core comprises active elements, such as air, oxygen, perfluorocarbons, sulfur hexafluoride, etc. [14,17,18,19,22,38,39,40,41]. The enclosed gas structure of NBs enables them to be echogenic when exposed to ultrasound, as there is a difference in the acoustic impedance of the gas core and the shell [14,18,19,21,22,24,30,32,40,42,43,44]. This difference enhances the overall ultrasound backscatter and contrast observed, which is why NB have been used as contrast agents in biomedical imaging for decades [18,21,24,30,42,44,45]. The outer membrane, or shell, is a monolayer constructed from biomolecules that encompass and protect the gas core [14,18,19,20,21]. The shell structure can be comprised of hydrophilic or amphiphilic biomaterials, such as proteins, lipids, phospholipids, polymers, and surfactants [14,18,19,20,21,25,28,29,30,32,41,46] (Figure 2). The constitution of the NB shell affects the degree of rigidity, stability, elasticity, biocompatibility, half-life, loading capacity, system clearance, and rate of diffusion of the inner gas to the surrounding environment [14,18,19,21,22,30,32,41,47]. NBs with shells comprised of lipids and polymers are often used in biomedical applications as they have higher loading capacities [18,19,26,28,29,32,44].

NBs with protein-based shells are highly stable, biodegradable, biocompatible, and possess an extended half-life [14,18,19,21]. The protein shells are manufactured by raising the temperature of the desired protein solution until the denaturation stage is reached, followed by combining the proteins to form an emulsion [18,19]. The newly denatured proteins encircle the selected gas to form a unilayer shell. Protein-based shells tend to be stiff and allow minimal gas exchange across the membrane [18,19]. Additionally, protein shells can be modified with the addition of polyethylene glycol (PEG) polymers, referred to as PEGylation, which improves the shelf life of the NBs as well as the overall stability [14,18,19,31,41].

Lipid-shelled NBs are used for biomedical applications because they are highly biodegradable and biocompatible, making them useful for preclinical and clinical objectives [18,19,21,23,28]. Lipids contribute to a more flexible shell membrane, enable effective gas exchange, and enhance acoustic resonance under sonography [18,21,23]. Phospholipids are also used in lipid-based shells as they can independently organize into monolayers around the gas core, along the interface between the gas core and liquid exterior [14,18,21,25,32]. The phospholipid shells consist of a hydrophilic head and hydrophobic tails, displaying amphiphilic characteristics, which enable gas and hydrophobic compounds to be effectively enclosed within the shells [14,18,21,25,28,46]. The construction of phospholipid shells can include the combination of base phospholipids with modified lipids, surfactants, and emulsifiers [14,17,18,19,20,21,25,31]. Surfactants are amphiphilic molecules that can help stabilize NBs by reducing the surface tension at the interfacial point [18,19,21,25,48].

Polymer-shelled NBs tend to have larger and thicker shells in comparison to protein and lipid shells [18,19]. The broader polymer shells enable NBs to possess an enhanced loading capacity for drug delivery, for both hydrophilic and hydrophobic drug compounds [18,19,21,46]. In general, polymeric shells are more stable when exposed to ultrasound fields, exhibiting more resistance to compression and expansion than other materials [18,21]. Many polymers, such as poly (glycolic acid) (PGA) and poly (lactic-co-glycolic acid) (PLGA), have been incorporated into shells because they contribute to improved stability, reproducibility, biocompatibility, purity, and shelf life [14,18,19,22,30]. Polymeric NBs can also be PEGylated similar to other shell types, which further upgrades the biocompatibility and functions to lower the immunogenic response [14,18,19,21,22,41].

NBs are generated via two overarching pathways, either involving the modification of pre-existing microbubbles or the formulation of novel NBs from scratch [14,17,20,43,46,49]. The main generation techniques include electrolysis, cavitation, acoustic, particle, hydrodynamic, optical cavitation, and membrane methods [14,19,20,25,38,39,42,43,45,47,50,51]. Additional synthetic techniques can be used to produce NBs, such as laser ablation, sonication, microfluidic devices, agitation, emulsification methods, etc. [18,19,26,28,30,43,45,46,47,49,51]. The electrolysis method involves splitting water into hydrogen and oxygen using electrochemical processes [17,20,38,39,45,47]. When a direct current is passed through the water, oxygen bubbles appear at the positive anode while hydrogen is released from the negative cathode [17,20,38,39,45,47,51]. The cavitation method consists of a rapid generation and collapse of vapor bubbles within a liquid, which occurs in areas of the liquid flow where the static pressure becomes smaller than the vapor pressure of the liquid [17,19,20,25,45,47,51]. The hydrodynamic cavitation method is performed within a system generator, where bubbles of water vapor form when the pressure in the narrowed spout decreases below the vapor pressure [17,19,20,45,47,51]. Similarly, the acoustic cavitation method utilizes ultrasound to drop the pressure and produce the energy necessary to cause oscillations leading to nucleation in a liquid [17,20,25,38,39,47,48,51]. Alternatively, the membrane method involves injecting the desired gas through apertures of varying sizes along a liquid medium to dissolve the gas into an aqueous state. The gas is compressed and directed through the pores of the membrane to produce a slurry of NBs with varying diameters [17,19,20,45,47].

The ability of the NB to function is linked to its size distribution, which is why accuracy in size measurement and distribution of NBs is crucial. The equipment used for measuring bubble size distribution and concentration has been reported in many studies. Some of these include coulter counter [52,53], cryo-electron microscopy (cryo-EM) [54,55], dynamic light scattering (DLS) [56,57], nanoparticle tracking analysis (NTA) [58,59,60], etc. The complete details of the equipment used for this purpose have been discussed elsewhere [30,61]. Due to their size, NBs can take advantage of the enhanced permeation and retention (EPR) effect observed in tumor tissues, which enables the selective accumulation and cellular perforation of nanoparticles [14,18,19,21,26,27,28,29,30,31,32,41,45].

In recent years, the combined effects of NBs with ultrasound have been extensively explored alongside existing cancer therapies like chemotherapy, immunotherapy and/or radiation therapy. These combination therapies are effective in destroying cancerous tumors.

## 3. Combinatory Effect of USNBs and Cancer Therapies

Chemotherapy remains one of the primary treatment options for several inoperable solid tumors. However, its efficacy remains questionable because of the side effects due to high drug toxicity [41,62,63]. Also, the poor stability and low water solubility make it harder for drugs to reach the tumor mass [64,65]. In recent years, the combination of drug-loaded NBs and ultrasound has been explored extensively [66,67,68,69]. This delivery method is known to reduce drug toxicity with increased stability, assuring a higher rate of drug reaching the targeted site [70,71]. Once the drug-loaded NBs are injected into the system, ultrasound waves are applied to cause the bubbles to burst so that the drug is released at the site of treatment [36,72]. Multiple methods have been proposed to improve the local drug accumulation in a tumor mass using NBs and ultrasound techniques [73].

A study conducted by Meng et al. used rabbit-bearing VX2 liver tumors to assess the efficacy of doxorubicin nanobubble (DOX-NB) followed by ultrasound exposure (DOX-NB+US) [74]. The result showed that the growth of the VX2 tumor was significantly suppressed by 76.7% with the combined treatment of DOX-NB+US compared to the control group. Furthermore, reduced tumor cell proliferation and increased cell death were observed in the combined group of DOX-NB+US [74]. A recent in vitro and in vivo study conducted by Yang et al. utilized nanobubbles carrying docetaxel (IR780-NBs-DTX) followed by ultrasound exposure for the treatment of pancreatic cancer [75]. Their in vitro, results showed a significant reduction in cell viability by 99.8 ± 2.1% as the concentration of IR780-NBs-DTX was increased. In vivo, results revealed almost disappearance of the tumor around 18 days after IR780-NBs-DTX treatment, showing the treatment to be highly effective in tumor control [75]. Another study by Nittayacharn et al. assessed the therapeutic efficacy and cellular uptake of doxorubicin using LS-174T in vitro and in vivo models [76]. The combination of doxorubicin-loaded nanobubbles (Dox-NBs) and ultrasound resulted in a greater decrease in cell viability compared to the control or single treatment group consisting of either chemotherapy, ultrasound plus chemotherapy, or NBs plus chemotherapy. Furthermore, the in vivo results indicated a higher accumulation of doxorubicin within tumors in the group receiving combined treatment of Dox-NBs and ultrasound as compared to the control or single treatment group [76]. Later to this, another in vitro study conducted utilized hydrophobic doxorubicin loaded C_3_F_8_ nanobubble (hDox-NB) or doxorubicin hydrochloride loaded-NBs (Dox.HCl-NBs) in human ovarian cancer cells (OVCAR-3) [77]. The therapeutic efficacy of this method was evaluated, showing a two-fold greater drug loading capacity with hDox-NBs compared to Dox.HCl-NBs that resulted in a higher decrease in cell viability [77]. A study by Batchelor et al. provided a detailed work on how the size and stability of NBs have an impactful role to play in the enhancement of drug delivery. It was demonstrated that the smaller size of bubbles has greater stability and enhanced dextran drug uptake [34].

Since hypoxia is an ongoing concern during the treatment of tumors, drug-loaded oxygen NBss have been explored extensively in the past few years. An in vivo and ex vivo study by Bhandari et al. investigated the therapeutic response of ultrasound-mediated oxygen nanobubbles (ONBs) in combination with mitomycin-C (MMC) (ONB-MMC) in bladder cancer [78]. Exposure of the tumor with ONB-MMC resulted in continuous oxygenation of the hypoxic tumor regions sensitizing the tumor area. This resulted in significant inhibition in tumor growth causing reduced tumor volume as compared to a control group. Furthermore, an ex vivo mouse bladder treated with ONB-MMC demonstrated lower staining for hypoxia-inducible factor 1 (HIF-1) and vascular endothelial growth factor (VEGF), indicating hypoxia and anti-angiogenesis might be potentially involved in reduced tumor volume [78].

A study by Yin et al. demonstrated that nanobubble-bearing siRNA (siRNA-NB) targeting the anti-apoptosis gene sirtuin 2 (SIRT2) upon ultrasound stimulation (SIRT2-NBs US (+)) caused increased C6 glioma cell death in vitro [79]. Similarly, an in vivo study conducted with mice bearing C6 glioma with exposure to SIRT2-NBs US (+) resulted in a significant reduction in tumor growth. Immunohistochemical analysis of the tumor section exposed to SIRT2-NBs US (+) revealed a marked increase in cell death confirmed using hematoxylin and eosin (H&E), caspase-3, and terminal deoxynucleotidyl transferase (TdT) dUTP Nick-End Labeling (TUNEL) assay. The study showed a greater therapeutic response following ultrasound combined with siRNA-NB [79]. The study was further extended utilizing nanobubble-encapsulated paclitaxel (PTX) and siRNA (PTX-NBs/siRNA) for the treatment of hepatocellular carcinoma (HCC) (in vitro and in vivo) [80]. The combination of ultrasound and PTX–NBs/siRNA caused an enhanced antitumor effect by inhibiting tumor growth in HCC-bearing animals [80].

There is enormous evidence that suggests the USNBs-mediated opening of blood-brain barrier (BBB) led to a significant increase in chemotherapeutic drug uptake [29,30,32]. However, limited studies are available on the impact of NB concentration and its stability on BBB opening and drug delivery. A study conducted by Cheng et al. [81] used different concentrations of NBs (dilutions of 1:1, 1:10, and 1:100) following focused ultrasound (FUS) treatment to access the bubble circulation time and opening of BBB in rats. Their result indicated that the undiluted bubbles (1:1) remained 10 min in circulation, whereas the circulation time for diluted bubbles (1:10 and 1:100) was reduced to 5–6 min. The successful opening of the BBB was reported with all the different concentrations of NBs [81]. The injection of NBs followed by FUS stimulation was reported to be reliable for BBB opening [81,82].

Numerous studies have demonstrated that NBs alone or in combination with ultrasound can be used as an effective treatment option [83]. A study conducted by Suzuki et al. demonstrated the effectiveness of combining USNBs in an in vivo model [83]. Experiments conducted with BALB/c mice bearing colon tumors when exposed to USNBs demonstrated significant suppression in tumor growth by 45% compared to control groups. A marked increase in necrosis was also reported in the USNBs group. The antitumor effects observed following this treatment were reported to be because of the participation of CD8^+^ T cells and not CD4^+^ T or natural killer cells [83]. Similarly, another study conducted in vitro and in vivo using a breast cancer model demonstrated enhanced therapeutic efficacy following USNBs [84]. Cells treated with USNBs showed a decrease in the number of viable cells by 17.3 ± 1.7% compared to the control group. Furthermore, the histology section obtained in vivo demonstrated a greater area of necrosis and fibrosis in the treated group as compared to the sham group or group treated with microbubbles and ultrasound that showed greater cellularity with no tissue disruption. The histology section obtained from different organs from sham and USNB groups showed no damage to these parts, indicating the USNB approach to be a safe and non-invasive treatment option for cancers [84].

The use of USNBs has been reported to increase the efficacy of immunotherapy. A study by Hu et al. utilized three different xenograft models, RM1 (prostate cancer), MC38 (colon cancer), and B16 (melanoma), to examine the effect of USNBs in combination with an anti-PD1 antibody [37]. Their result indicated an increase in tumor necrosis, release of damage-associated molecular pattern (DAMP), and tumor antigen presentation, resulting in inhibition of tumor growth ultimately leading to tumor sensitization. Thus, an increase in antitumor immunity following USNB was reported in this study [37].

The role of USNBs in enhancing the effectiveness of radiation therapy has also been explored. An in vivo study conducted by Hysi et al. utilized NBs in combination with radiation therapy (8 Gy) for the treatment of prostate cancer [13]. It was demonstrated that the combined treatment of USNBs and radiation therapy caused a significant increase in cell death by 40%, alongside a decrease in oxygen saturation by 18% and a reduction in vessel counts by 50% compared to the control group. Furthermore, a reduction in tumor volume by 70% was reported in groups treated with USNBs and radiation therapy. A comparison made between the data obtained from the USNB and USMB group suggested that the tumor response following NBs and radiation therapy was higher compared to the microbubbles and radiation therapy group [13].

The summary of the combined effects of USNBs and existing cancer therapies (chemotherapy, immunotherapy, and/or radiation therapy) is presented in Table 1.

Several preclinical studies suggest that the antivascular effects and bioeffects observed upon exposure to microbubbles and ultrasound are due to the involvement of the ASMase/ceramide pathway [4,85]. Microbubble destruction upon ultrasound causes perturbation of endothelial cell lining that causes the release of ASMase, an enzyme found 20-fold higher in endothelial cells [86]. The generated ASMase further releases ceramide, a cell death signaling molecule that leads to the destruction of tumor vasculature resulting in overall suppression of tumor growth and increased survival rates [11,87]. Evidence suggests that blocking the activation of ASMase or generation of ceramide using basic fibroblast growth factor (bFGF) or sphingosine-1-phosphate (S1P) attenuates the ASMase/ceramide-mediated antitumor effects [11,12,88,89,90]. Cancer therapy such as radiation therapy and/or chemotherapy is known to elevate the production of ASMase/ceramide, attributing to enhanced tumor vascular damage and tumor cell death [91,92,93]. Thus, USMB-induced vascular endothelium damage due to the activation of the ASMase and ceramide pathway is known to be the main regulatory mechanism in ultrasound-based microbubble therapy [11,12,94]. This mechanism has not been tested for nanobubble-based therapy; however, it can be anticipated that a similar phenomenon might be involved when using NBs and ultrasound.

**Table 1:** Summary of effects of USNB combined with existing cancer therapies. Abbreviation: Bax; (Bcl-2)-associated X, Bcl-2; B-cell lymphoma 2; CD86; cluster of differentiation 86, CD80; cluster of differentiation 80, HIF-1; hypoxia-inducible factor 1, IFN-γ; interferon-gamma, IL-2; interleukin-2, OVA; ovalbumin, PTT; photothermal therapy, TNF-α; tumor necrosis factor alpha, USNBs; ultrasound-stimulated nanobubbles, VEGF; vascular endothelial growth factor.

	Experimental Model	Treatment Regimen	Ultrasound Parameters	Cellular/Tumor Response	Reference
**Chemotherapy**					
	Human ovarian cancer cells (OVCAR-3) in vitro	USNB + Chemotherapy (doxorubicin)	Transducer frequency: 1 MHz Intensity: 1.7 W/cm^2^ Duty cycle: 100% for 1 min	Improved drug loading capacity and acoustic signal, decrease in cell viability	[77]
	Human lung cancer cells (A549) in vitro	USNB (survivin-siRNA bound) + Chemotherapy (paclitaxel)	Transducer frequency: 3 MHz E = 449 J, 5 min	Decreased survivin expression, increased siRNA delivered to target region, increased apoptosis	[95]
	Human colorectal cancer cells (LS-174T) in vitro and in vivo (mice)	USNB + Chemotherapy (doxorubicin)	Transducer frequency: 3 MHz Intensity: 2 W/cm^2^ Duty cycle: 20% for 1 min	Increased targeted drug accumulation and intracellular uptake, decreased cell viability	[76]
	Murine bladder cancer cells (MB49) in vitro and in vivo (mice)	USNB (Oxygen-bound) + Chemotherapy (mitomycin-C)	Transducer frequency: 40 MHz Duty cycle: 20% and 100%	Reduced tumor progression rates, increased cell death and enhanced re-oxygenation of hypoxic tumor regions, decreased level of HIF-1 and VEGF expression	[78]
	Human liver cancer cells (HepG2) in vitro and in vivo (mice)	USNB (siRNA-bound) + Chemotherapy (paclitaxel)	Transducer frequency: 1 MHz Pressure: 500 kPa Duty cycle: 50%	Enhanced drug and siRNA codelivery, cell apoptosis, reduced tumor volume, higher animal survival rates	[80]
	Human pancreatic cancer cells (Mia-Paca2) in vitro and in vivo (mice)	USNB + PTT + Chemotherapy (docetaxel)	Transducer frequency: 7.5 MHz Intensity: 2.5 W/cm^2^ PTT: 808 nm (1 W/cm^2^, 210 s)	Improved tumor tar-geting rates, increased apoptosis, reduction in tumor size and cellular proliferation	[75]
	Liver cancer (VX2) in vitro and in vivo (rabbits)	USNB + Chemotherapy (doxorubicin)	Transducer frequency: 1 MHz Intensity: 2 W/cm^2^	Increased drug release decreased growth rate, reduced proliferation, and increased apoptosis, greater survival rates	[74]
**Immunotherapy**					
	Murine prostate (RM1, RM1-OVA), colon (MC38, MC38-OVA) and melanoma (B16) cancer cells in vitro and in vivo (mice)	USNB + Immunotherapy (anti-PD1)	Transducer frequency: 1 MHz Intensity: 1 W/cm^2^	Decreased tumor growth and metastasis, increased immune response and immune memory	[37]
	Murine liver cancer cells (H22) in vivo (mice)	USNB + Immunotherapy (sPD-1 and Ce6)	Transducer frequency: 1.1 MHz Intensity: 1.8 W/cm^2^ Duty cycle: 50%	Decreased Bcl-2 mRNA, increased expression of Bax, CD80, CD86, IFN-γ, TNF-α, and IL-2, increased tumor apoptosis and necrosis	[96]
**Radiation** **Therapy**					
	Human prostate cancer cells (PC3) in vivo (mice)	USNB + Radiation therapy (8 Gy)	Transducer frequency: 500 kHz Pressure: 570 kPa Duty cycle: 0.24% or 720 ms	Increased cell death, reduced vessel counts, decreased oxygen saturation, reduced tumor size	[13]

## 4. Clinical Trials

In recent years, NBs have drawn the attention of scientists in the field of cancer treatment; however, their clinical use has remained relatively low. At present, there exist no clinical trials conducted using NB-based therapy. An in situ study by Huynh et al. demonstrated for the first time that the porphyrin microbubbles can be converted to NBs by ultrasound, suggesting a possibility of using this method in the future for increased drug permeability and retention effect. [33]. Later on, Pellow et al. provided a detailed study on the extravasation of NBs to the targeted tumor sites [31]. They incorporated a phantom scattering model and an in vivo model (mice with a green fluorescent protein (GFP) tagged human FaDu squamous cell carcinoma cells) to show that NBs retained their stability for around 20 min, circulating in the vasculature. Following NBs injection and ultrasound exposure, higher NBs extravasation was observed over time preferably in the tumor area as compared to control groups. Furthermore, upon initial destructive probe pulse, high cavitation was observed in tumors while no cavitation was observed in the healthy control groups, indicating that the intact NBs were absent in the control groups. The sonication of 2 min resulted in a higher release of fluorescent materials seen more in tumors as compared to a healthy control group. This observation might be of great importance when considering drug delivery to solid tumors using NBs and ultrasound. Several bioeffects such as vascular disruption, vascular shutdown and sonoprinting were also reported following the implementation of USNBs [31]. These studies serve as a gateway for the implication of NBs in clinical settings. Compared to NBs, multiple clinical trials have been conducted using ultrasound and microbubbles. USMBs combined with either chemotherapy and/or radiation therapy has been shown to have less to no severe toxicity or side effects in clinical settings. The combination of USMB and these cancer therapies have been successfully implemented in treating breast cancer [97,98], bile duct cancer [99], gliomas [100], hepatocellular carcinoma [101], head and neck squamous (NCT04431648), liver cancer [102,103], ovarian cancer [97], and pancreatic carcinoma [104]. These studies substantiate enormous potential of nanobubbles-ultrasound based therapy for future clinical trials.

## 5. Conclusions

The use of USNBs with existing cancer therapies is a promising therapeutic approach for treating various types of cancers. However, its application in clinical use still requires an efficient approach to target tumors which remains a major challenge. Currently, in preclinical settings, USNBs are given alongside chemotherapy, immunotherapy, and/or radiation therapy. Ultrasound-mediated chemotherapeutic delivery using NBs has been demonstrated to increase cell/vascular membrane permeability, resulting in greater drug distribution as well as penetration of drug concentrations in the interstitial tissue. Furthermore, through mechanical or thermal effects of USNBs, the release of antigens from tumor cells promotes antigen presentation and T cells recognition resulting in the killing of tumor cells. Moreover, USNB-mediated cavitation is known to increase the effectiveness of radiation therapy. The biological effects of USNBs in combination with radiation therapy result in tumor vasculature damage, inducing anticancer effects. Despite the growing evidence that suggests USNBs may potentially be an adjuvant treatment option for treating various tumors, their implication in clinical settings remains largely elusive. Further research is needed in the areas of NBs, with a strong emphasis on their potential side effects and long-term benefits.
